# Comorbidity of Dengue and Hereditary Spherocytosis in an 18-Year-Old Patient: A Case Report

**DOI:** 10.7759/cureus.107168

**Published:** 2026-04-16

**Authors:** Rogelio I Becerra-Carrillo, César Mendoza-Maldonado, Horacio Rendón-Aguilar, Mariana Torres-Hernández, Luis Ochoa-Ramírez

**Affiliations:** 1 Biomedicine, Faculty of Biology, Universidad Autonoma de Sinaloa, Culiacán, MEX; 2 Biomedical Sciences, Faculty of Chemical and Biological Sciences, Universidad Autonoma de Sinaloa, Culiacán, MEX; 3 Hematology, Servicios de Salud del Instituto Mexicano del Seguro Social para el Bienestar, Hospital General Culiacán, Culiacán, MEX; 4 Internal Medicine, Servicios de Salud del Instituto Mexicano del Seguro Social para el Bienestar, Hospital General Culiacán, Culiacán, MEX; 5 Genomic Medicine, Servicios de Salud del Instituto Mexicano del Seguro Social para el Bienestar, Hospital General Culiacán, Culiacán, MEX

**Keywords:** dengue infection, hemolytic anemia, heriditary spherocytosis, hyperbilirubinemia, thrombocytopenia

## Abstract

Hereditary spherocytosis (HS) is one of the most common non-immune hemolytic anemias, and it’s characterized by defects in erythrocyte membrane proteins that result in increased red blood cell fragility and hemolysis. Dengue is an acute viral infection frequently associated with hematologic alterations, including thrombocytopenia and plasma leakage. The coexistence of dengue infection and HS has rarely been reported in the literature. In this report, we describe the case of an 18-year-old male with a history of HS, diagnosed during childhood, who presented with a five-day history of fever and headache. Serologic testing confirmed dengue infection. Laboratory studies revealed severe anemia and thrombocytopenia. Peripheral blood smear showed anisocytosis and spherocytes. Imaging studies demonstrated hepatomegaly, splenomegaly, gallbladder wall edema, and free intraperitoneal fluid. The patient improved with supportive management (such as intravenous Hartmann solution and the transfusion of two red blood cell concentrates) and was discharged without complications. This case highlights a rare comorbidity that exacerbates the hematologic abnormalities in patients with dengue infection.

## Introduction

Hereditary spherocytosis (HS) is a hematological disorder that is considered one of the most common non-autoimmune hemolytic anemias. It is caused by genetic mutations that lead to structural abnormalities of the red blood cell membrane, resulting in the formation of hyperchromic, smaller, and more fragile erythrocytes compared with normal red cells [1,2].

The pathophysiology of HS is primarily attributed to an ineffective cell membrane that fails to function adequately as a protective barrier for blood cells [3]. This alteration results in decreased membrane stability and increased erythrocyte fragility. Clinically, HS is characterized by manifestations such as anemia, jaundice, and splenomegaly [3,4].

On the other hand, dengue disease is an acute febrile viral infection transmitted by mosquito bites, commonly observed in tropical and subtropical regions, and is associated with a wide spectrum of hematological manifestations ranging from fever to severe complications, including hemorrhagic fever, hepatic injury, and plasma leakage [5].

Among the risk factors for the exacerbation of dengue symptoms are the presence of underlying hematological disorders, such as hemolytic anemia and thrombocytopenia [6]. Therefore, the comorbidity between HS, the most common hemolytic anemia, and dengue infection may increase the likelihood of developing a more severe presentation of the infectious disease or exacerbate the manifestations of the underlying hematological condition.

In this case report, we describe the clinical management of a patient diagnosed with dengue and classified according to the World Health Organization (WHO) 2009 criteria [7] as dengue with warning signs, who had a history of HS diagnosed since the age of 6 and was already under treatment of 0.4 mg of folic acid per day, a clinical combination that has only been reported one time in the literature [8].

## Case presentation

An 18-year-old male presented to the emergency room (ER) with a five-day history of fever reaching up to 39.5°C and headache. Previously, the patient sought medical attention at another healthcare facility, where he received intravenous fluids (IVF) and was subsequently discharged from home.

The patient later experienced worsening symptoms, prompting him to visit a private laboratory where serological testing for dengue was performed, showing positive NS1 antigen, positive IgG, and negative IgM.

In the following days, the patient’s symptoms continued to progress. Due to this clinical deterioration, a complete blood count was performed at a private laboratory, revealing low hemoglobin (5.9 g/dL), hematocrit (15%), erythrocyte count (1.87×10^6^/µL), and platelet count (79×10^3^/µL). Following these findings, the patient was admitted to the Culiacan General Hospital ER, where further diagnostic evaluation was performed.

At hospital admission, laboratory studies revealed severe anemia, with erythrocyte count, hemoglobin, and hematocrit values below the reference ranges. Thrombocytopenia and red cell distribution width parameters (RDW-CV and RDW-SD) elevations were also documented. Leukocyte, mean corpuscular hemoglobin concentration (MCHC), and mean corpuscular volume (MCV) were within reference ranges.

Liver function revealed predominantly indirect hyperbilirubinemia. Additionally, elevations in transaminases were observed, including aspartate aminotransferase (AST) and alanine aminotransferase (ALT), as well as in lactate dehydrogenase (LDH) levels.

Coagulation studies revealed a capillary refill time of 3 seconds and prolonged prothrombin time, as well as prolonged activated partial thromboplastin time (Table 1).

**Table 1:** Laboratory findings during the patient’s clinical course *Twelve days after medical discharge

Parameter	Hospital admission	Day 1	Day 2	Day 3	Follow-up*	Reference range
Erythrocytes (×10^6^/µL)	1.98	-	2.43	2.63	4.03	4.20 - 6.30
Hemoglobin (g/dL)	5.6	-	7.0	7.8	12.1	12.00 - 18.00
Hematocrit (%)	16.8	-	20.7	24.7	-	37.00 - 51.00
Platelets (×10^3^/µL)	95	-	108	123	261	150.00 - 450.00
Red cell distribution width (RDW-CV) (%)	18.2	-	17.4	24.5	15.2	13.00 - 16.00
Red cell distribution width (RDW-SD) (fL)	55.9	-	50.8	61.1	-	39.00 - 48.00
Prothrombin time (PT) (Seg.)	-	20.3	-	-	-	10 - 16
Partial thromboplastin time (PTT) (Seg.)	-	53.6	-	-	-	25 - 45
Lactate dehydrogenase (LDH) (U/L)	954.93	841	874.32	-	-	240 - 480
C-reactive protein (CRP) (mg/dL)	-	-	34.77	-	-	0 - 6
Blood urea nitrogen (BUN) (mg/dL)	20.25	19.53	18.62	-	-	7.00 - 18.00
Total bilirubin (mg/dL)	5.34	4.54	2.73	-	7.2	0.00 - 1.00
Direct bilirubin (mg/dL)	1.04	0.91	0.75	-	0.4	0.10 - 0.40
Indirect bilirubin (mg/dL)	4.30	3.63	1.98	-	6.8	0.00 - 0.80
Aspartate aminotransferase (AST)(U/L)	125.85	119.58	98.45	-	-	17.00 - 59.00
Alanine aminotransferase (ALT) (U/L)	136.15	125.94	114.03	-	-	21.00 - 55.00
Mean corpuscular volume (MCV) (fL)	-	-	85.4	94.0	84	80.00 - 97.00
Mean corpuscular hemoglobin concentration (MCHC) (g/dL)	-	-	33.5	31.5	35.7	31.00 – 37.00

During the hospital course, follow-up studies were performed, showing persistence of anemia and thrombocytopenia, as well as sustained elevation of LDH and abnormalities in liver function tests. As a response to the patient’s sustained low hemoglobin levels (5.6 g/dL), two red blood cell concentrates were transfused, improving the hemoglobin levels to 7.0 g/dL. In a subsequent complete blood count, hemoglobin remained at 7.0 g/dL with platelet counts at 108×103/µL. Inflammatory markers showed a significant elevation of high-sensitive C-reactive protein (34.77 mg/dL), and the direct COOMBS test was negative.

**Figure 1:**
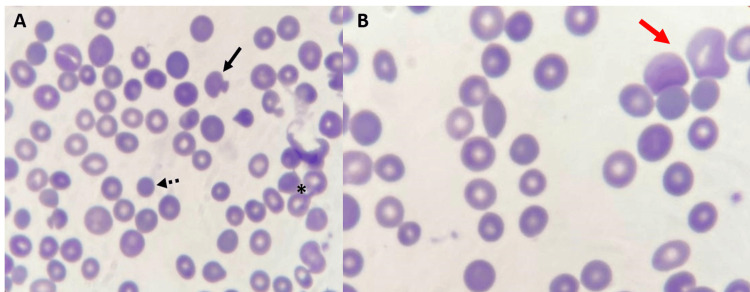
Wright-stained peripheral blood smear of the patient. In image A, the presence of spherocytes (dashed arrow), pincered cells (arrow), and marked anisocytosis (asterisk) can be observed (100x magnification). There is also the presence of stress reticulocytes, indicating polychromasia in B (red arrow). Howell-Jolly bodies were not found.

Regarding studies performed to support the diagnosis of HS, the peripheral blood smear demonstrated anisocytosis, as well as the presence of pincered cells and spherocytes (Figure 1), characteristic findings in patients with HS. Additionally, reticulocyte testing revealed a markedly elevated relative count of 9.11% and an absolute count of 295,164/µL, consistent with increased erythropoietic activity. Furthermore, the osmotic fragility test demonstrated increased red blood cell fragility, with 50% hemolysis occurring at a sodium chloride concentration of 0.57%.

**Figure 2:**
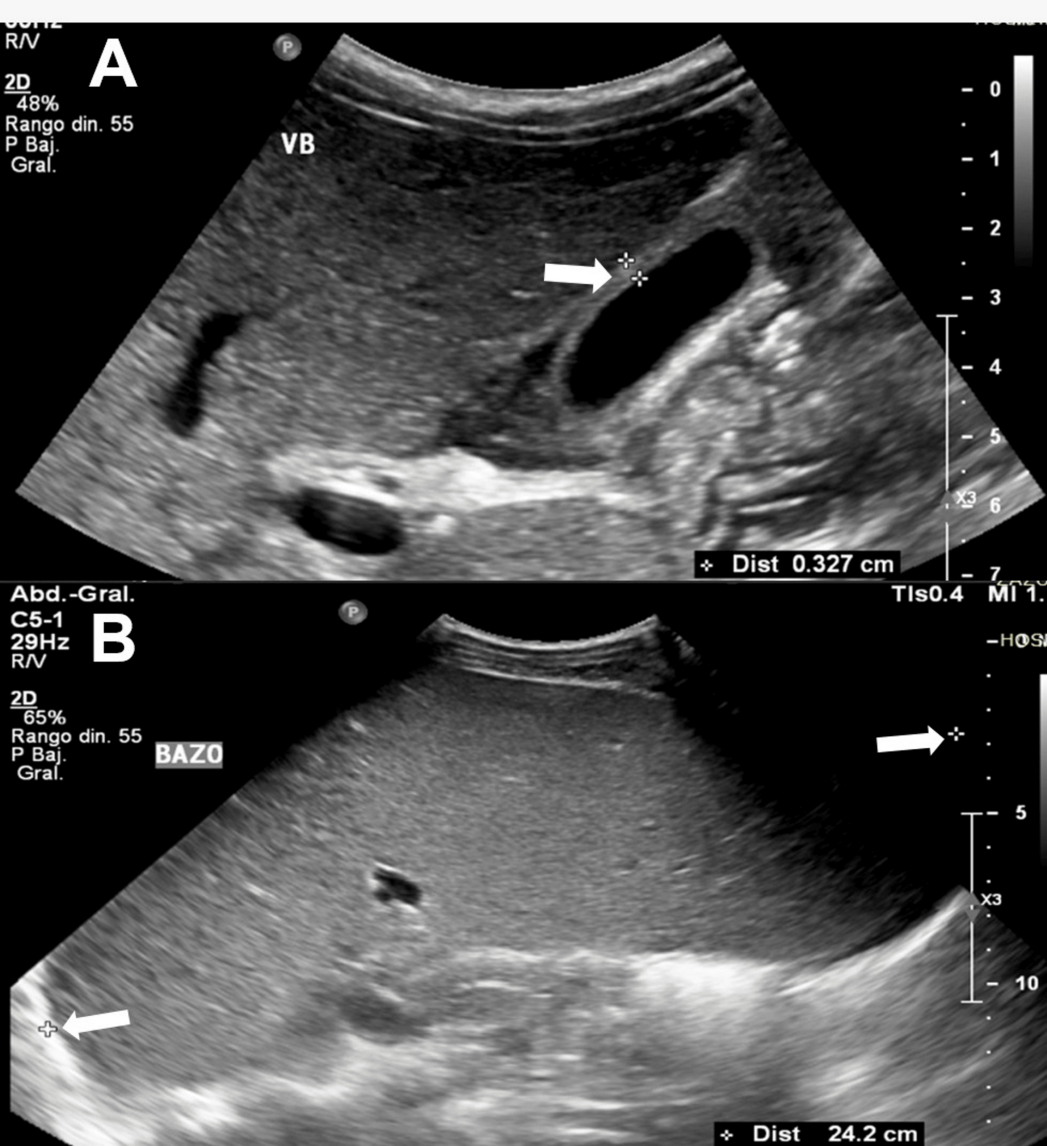
Abdominal ultrasound showing the patient’s gallbladder and spleen. The presence of reactive gallbladder wall edema is observed (arrow in A), as well as marked splenomegaly (arrows in B).

Within the imaging studies, the abdominal ultrasound revealed marked splenomegaly, as well as a reactive gallbladder wall edema (Figure 2).

**Figure 3:**
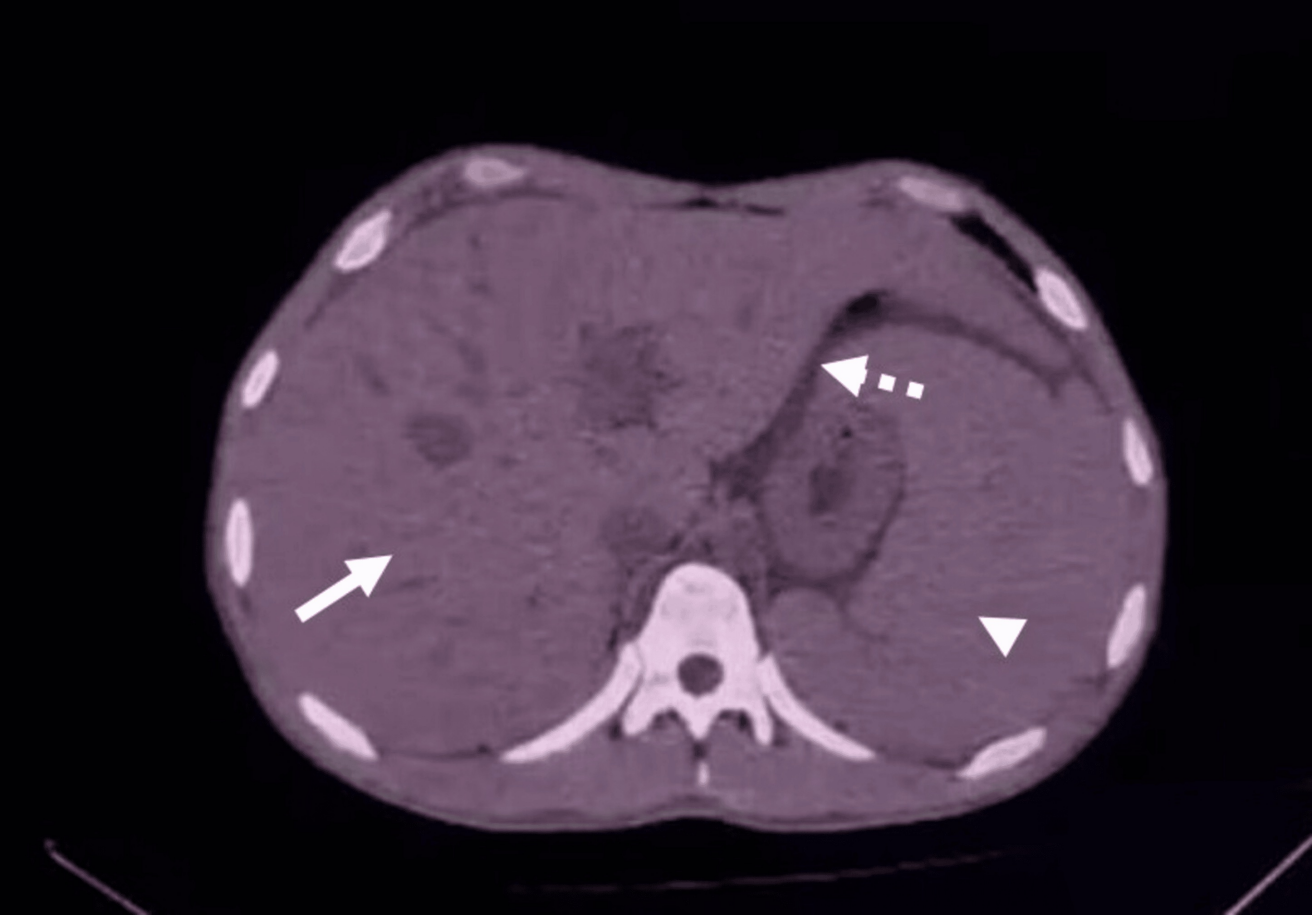
Patient’s transversal abdominopelvic tomography showing the abdominal cavity. The presence of splenomegaly (arrowhead), hepatomegaly (arrow), and free intraperitoneal fluid (dashed arrow) is demonstrated.

Computed tomography scans complemented the clinical evaluation, allowing for the exclusion of additional causes of the abdominal findings and confirming the presence of hepatomegaly, splenomegaly, and free intraperitoneal fluid (Figure 3). Overall, the imaging findings (hepatomegaly, splenomegaly, gallbladder wall edema, and ascites) are consistent with plasma leakage and systemic inflammatory response, which are recognized features of dengue infection.

After the third day of hospitalization, the patient was discharged from the medical unit without further complications. The final diagnosis was dengue without warning signs in a patient with underlying spherocytosis. Twelve days after his medical discharge, the patient underwent follow-up studies, where his values approached the reference values, but he continued to show characteristics of his hematological condition.

## Discussion

In the present case, the patient’s clinical course, characterized by the persistence of hematological abnormalities despite being initially classified as dengue without warning signs, suggests that the coexistence of both conditions may have influenced the severity of the presentation. The combination of a viral infection associated with thrombocytopenia and hepatic alterations, together with chronic hemolytic anemia such as hereditary spherocytosis, represents a complex clinical scenario that may complicate timely clinical management.

From a laboratory perspective, the serological test results suggest a secondary dengue infection. The absence of Howell-Jolly bodies on the peripheral blood smear suggests preserved splenic function. However, this finding does not exclude increased splenic activity, particularly in the context of HS. On the other hand, hyperbilirubinemia with a predominance of indirect bilirubin was found, accompanied by markedly elevated LDH levels. These findings are consistent with an ongoing intravascular hemolytic process, as evidenced by negative Coombs, and have been widely described in patients with HS [9,10]. Additionally, the coagulation parameters were altered, but in this case, despite the presence of thrombocytopenia and these altered values, the patient did not develop significant bleeding manifestations. This may suggest that, although laboratory abnormalities were present, they did not translate into clinically relevant hemorrhagic manifestations. Nevertheless, the coexistence of these conditions warrants close monitoring, as their combination may increase the risk of bleeding, particularly in patients with additional hematological comorbidities.

Imaging findings such as hepatomegaly, splenomegaly, gallbladder wall edema, and the presence of free intraperitoneal fluid have been previously described in dengue infection [10]. The presence of splenomegaly is relevant given the patient’s underlying HS. The spleen plays a central role in the pathophysiology of HS, as it is the primary site of erythrocyte destruction and splenic sequestration [11]. From this point of view, dengue infection is characterized by a robust systemic inflammatory response with increased levels of pro-inflammatory cytokines, which contribute to endothelial dysfunction and immune activation [12]. In this context, splenic involvement may be relevant. Dengue has been associated with splenic congestion and increased macrophage activity, which may enhance the clearance of abnormal erythrocytes. In patients with HS, red blood cells are more fragile and prone to sequestration within the splenic cords [13]. Therefore, the coexistence of dengue-related inflammation and HS may create a synergistic effect, which heightens splenic phagocytic activity and congestion, accelerating extravascular hemolysis. This mechanism could explain the persistence and severity of hematological abnormalities observed in this patient.

In endemic settings, differentiating dengue-related hematologic alterations from hemolytic exacerbations in patients with HS is essential for appropriate management. While dengue is typically associated with leukopenia, thrombocytopenia, and mild to moderate transaminase elevation, it does not usually cause significant hemolysis [5]. In contrast, HS-related hemolytic crises are characterized by reticulocytosis, elevated LDH, and indirect hyperbilirubinemia [1]. A peripheral blood smear is particularly informative, as the presence of spherocytes supports ongoing extravascular hemolysis. Although increased MCHC is classically associated with HS, it may remain within normal ranges, as observed in this case, and should not be used in isolation. Therefore, an integrated approach combining red blood cell indices, hemolysis markers, and peripheral morphology is essential to distinguish these overlapping conditions and guide clinical decision-making.

Although the patient had a favorable outcome without major complications, one previous report described a fatal outcome [8]. In that report, the patient was diagnosed with dengue fever and classified as dengue with warning signs, requiring transfusion of two units of whole blood, nine units of packed red blood cells, 22 units of fresh frozen plasma, and 10 units of platelet concentrate, highlighting the potential risk faced by patients with both conditions.

This report has several limitations that should be acknowledged. First, as a single case report, findings cannot be generalized to broader populations. Second, although the patient had a previously established diagnosis of HS, additional confirmatory tests, such as eosin-5’ maleimide (EMA) binding assay or genetic testing, were not performed during hospitalization, which may limit the diagnostic characterization of the condition. However, the diagnosis was supported by clinical history, peripheral blood smear findings, reticulocyte counting, and increased osmotic fragility. As to the third limitation, the case presentation may be confused with other negative Coombs hemolytic anemias, such as G6PD deficiency-related hemolytic anemia [14].

Additionally, dengue serotyping was not performed, which may limit the determination of the case complication depending on the serotype causing the infection. Moreover, there was also a lack of certainty on whether the infection was primary or secondary. 

## Conclusions

The coexistence of dengue infection and hereditary spherocytosis represents a rare and potentially underrecognized clinical scenario. This case highlights the importance of considering underlying hematological disorders in patients with dengue, as their presence may contribute to the exacerbations of hematological-based disorders and influence clinical monitoring. Early recognition of this association is essential, as it may guide closer hematologic monitoring, including serial hemoglobin, platelet counts, and markers of hemolysis. In addition, clinicians should be aware of the potential for exacerbation of anemia and carefully assess bleeding risk in the context of dengue-associated thrombocytopenia and coagulopathy. Timely identification of this comorbidity may support appropriate clinical surveillance and individualized management strategies in affected patients.
